# Exploiting three-dimensional human hepatic constructs to investigate the impact of *rs174537* on fatty acid metabolism

**DOI:** 10.1371/journal.pone.0262173

**Published:** 2022-01-20

**Authors:** L. Madison Kirk, Charlotte Mae K. Waits, Alexander C. Bashore, Beverly Dosso, Allison K. Meyers, Antonio C. Renaldo, Thomas J. DePalma, Kelli N. Simms, Nathaniel Hauser, Chia-Chi Chuang Key, Charles E. McCall, John S. Parks, Susan Sergeant, Carl D. Langefeld, Aleksander Skardal, Elaheh Rahbar

**Affiliations:** 1 Department of Biomedical Engineering, Wake Forest School of Medicine, Winston-Salem, North Carolina, United States of America; 2 Virginia Tech – Wake Forest University, School of Biomedical Engineering and Sciences, Blacksburg, Virginia, United States of America; 3 Division of Cardiology, Department of Medicine, Columbia University Irving Medical Center, New York, New York, United States of America; 4 Department of Internal Medicine, Section of Molecular Medicine, Wake Forest School of Medicine, Winston-Salem, North Carolina, United States of America; 5 Department of Integrative Physiology & Pharmacology, Wake Forest School of Medicine, Winston-Salem, North Carolina, United States of America; 6 Department of Microbiology and Immunology, Wake Forest School of Medicine, Winston-Salem, North Carolina, United States of America; 7 Department of Biomedical Engineering, The Ohio State University, Columbus, Ohio, United States of America; 8 Department of Biochemistry, Wake Forest School of Medicine, Winston-Salem, North Carolina, United States of America; 9 Department of Biostatistics and Data Science, Wake Forest School of Medicine, Winston-Salem, North Carolina, United States of America; 10 Center for Precision Medicine, Wake Forest School of Medicine, Winston-Salem, North Carolina, United States of America; Universite du Quebec a Montreal, CANADA

## Abstract

The Modern Western Diet has been associated with the rise in metabolic and inflammatory diseases, including obesity, diabetes, and cardiovascular disease. This has been attributed, in part, to the increase in dietary omega-6 polyunsaturated fatty acid (PUFA) consumption, specifically linoleic acid (LA), arachidonic acid (ARA), and their subsequent metabolism to pro-inflammatory metabolites which may be driving human disease. Conversion of dietary LA to ARA is regulated by genetic variants near and within the fatty acid desaturase (FADS) haplotype block, most notably single nucleotide polymorphism *rs174537* is strongly associated with FADS1 activity and expression. This variant and others within high linkage disequilibrium may potentially explain the diversity in both diet and inflammatory mediators that drive chronic inflammatory disease in human populations. Mechanistic exploration into this phenomenon using human hepatocytes is limited by current two-dimensional culture models that poorly replicate *in vivo* functionality. Therefore, we aimed to develop and characterize a three-dimensional hepatic construct for the study of human PUFA metabolism. Primary human hepatocytes cultured in 3D hydrogels were characterized for their capacity to represent basic lipid processing functions, including lipid esterification, de novo lipogenesis, and cholesterol efflux. They were then exposed to control and LA-enriched media and reproducibly displayed allele-specific metabolic activity of FADS1, based on genotype at *rs174537*. Hepatocytes derived from individuals homozygous with the minor allele at *rs174537* (i.e., *TT*) displayed the slowest metabolic conversion of LA to ARA and significantly reduced FADS1 and FADS2 expression. These results support the feasibility of using 3D human hepatic cultures for the study of human PUFA and lipid metabolism and relevant gene-diet interactions, thereby enabling future nutrition targets in humans.

## Introduction

The Modern Western Diet (MWD) has been associated with the rising prevalence of metabolic disorders, including diabetes, obesity, cardiovascular, and chronic inflammatory diseases [1–3]. One possible reason for the rise in metabolic disorders is the doubling of omega-6 (n-6) polyunsaturated fatty acid (PUFA) intake due to increased consumption of refined vegetable oils, resulting in high levels of essential dietary n-6 PUFA, linoleic acid (LA, 18:2n-6) [1,4–6]. Recent reports indicate that current consumption of LA is ~13–16 g per day, translating to about 6–8% of the US daily energy intake [7]. These extremely high LA levels are concerning as they have been linked to a variety of inflammatory diseases and cancer. In fact, latest nutritional guidelines encourage reducing LA intake to 1–2% of total energy, comparable to that found in the Mediterranean diet [8].

Nevertheless, there continues to be debate over the optimal dietary levels of PUFAs and their role in preventing or attenuating inflammatory diseases [9–11]. Dietary PUFAs are metabolized predominantly in the liver through a series of fatty acid desaturation and elongation steps via the fatty acid desaturase (FADS) and elongation of very long chain (ELOVL) fatty acid enzymes. The enzymatic activity of FADS and ELOVL are regulated by genetic variants corresponding to the *FADS* and *ELOVL* genes, respectively. Specifically, LA is metabolized into long chain PUFAs: arachidonic acid (ARA, 20:4n-6) and adrenic acid (22:4n-6), which are precursors to a series of proinflammatory and pro-thrombotic metabolites [1,6,7]. In a recent prospective dietary supplementation study, the metabolic conversion of LA to ARA in humans was shown to be strongly associated with genotype at *rs174537*, a single nucleotide polymorphism (SNP) located downstream of *FADS1* on chromosome 11 [12]. In fact, SNPs *rs174537* and *rs174550* (which are in perfect linkage disequilibrium (LD)) have been shown to affect both the metabolic conversion of LA to ARA, and the production of lipoxygenase-derived inflammatory oxylipins [13–16]. Individuals homozygous with the minor allele at *rs174537* (i.e., *TT*) display the slowest conversion of dietary PUFAs, nearly half the rate compared to homozygous major allele carriers (i.e., *GG*). Additionally, *TT*’s are often considered to be deficient in long chain PUFAs, unlike GG’s that have significantly higher circulating and tissue levels of ARA [14,16,17]. There is growing evidence that these SNPs, and potentially others in high LD, could explain differential responses not only to dietary PUFAs, but also their impact on inflammation. However, the mechanistic study of these effects within the liver remains difficult due to the lack of reliable human *in vitro* models.

Three-dimensional (3D) tissue engineered platforms and engineered microphysiological systems of the liver have demonstrated promise for the study of human liver function. Over the past decade, 3D liver spheroids, organoids and organ-like constructs have been successfully developed and used to study the effects of drug metabolism and chemical/toxin clearance. In fact, the culturing of primary human hepatocytes (PHH) in 3D spheroids and/or organoid based systems have been shown to replicate human liver function *in vitro* [18,19]. In these models, hepatocytes retain their cell-to-cell contacts, viability, functionality and even interactions with the extracellular matrix (ECM), when cultured in hydrogels or scaffold-based systems. While such organ-like cultures and liver spheroids have been successfully used for the study of drug metabolism, there is unexplored potential for investigating lipid and PUFA metabolic applications within this *in vitro* human liver platform. Here, we aimed to characterize 3D hepatic constructs for the study of PUFA and lipid metabolism, with a focus on the effects of *rs174537* on PUFA metabolism and *FADS* expression. We characterized rates of lipogenesis and cholesterol efflux. We also compared constructs exposed to LA vs. vehicle controls and demonstrated allele-specific metabolic activity with respect to *rs174537*, with hepatocytes derived from *TT* individuals displaying the lowest levels of ARA and FADS1 activity. The 3D human hepatic culture system was able to replicate *in vivo* phenomena related to fatty acid and lipid metabolism. Collectively, our results illustrate the feasibility of exploiting 3D human hepatic culture systems for the study of future gene-diet interactions. This *in vitro* human hepatocyte model can provide great mechanistic insights into the functional variants regulating PUFA and lipid metabolism, as well as offer new targets for delivering precision nutrition.

## Materials and methods

### Primary human hepatocytes

Cryopreserved primary human hepatocytes (PHHs) were obtained from Gibco (ThermoFisher Scientific, Waltham, MA) through Wake Forest’s Comprehensive Cancer Center Tumor Tissue and Pathology Shared Resource (n = 12). An additional two batches of commercially available PHH were purchased from Lonza (Basel, Switzerland) (n = 2). Because race and ethnicity influence lipid and fatty acid biosynthesis and metabolic conversion capacities, this study was limited to Caucasian donors only [4,20]. Table 1 shows vendor provided cell demographics and origin. Donors experienced traumatic deaths, but otherwise were healthy with an average age of 47 years. Cells were counted using a hemocytometer and 0.04% Trypan Blue Stain (Cat#15250061). All media, materials, and supplements were obtained from ThermoFisher Scientific, unless otherwise noted. PHHs were thawed according to the vendor’s recommendations and resuspended in Williams-E media (Cat#12551032) supplemented with the Primary Hepatocyte Thawing and Plating Supplements (Cat#CM3000), containing fetal bovine serum, dexamethasone, penicillin-streptomycin, human recombinant insulin, GlutaMAX^™^, and HEPES. Media was changed daily; conditioned media was stored in 250 μl aliquots at -80°C for future analysis.

**Table 1:** Overview of primary human hepatocytes (PHHs) used in the experiments.

Vendor	Donor	Gender	Age	BMI	*rs174537*
**Gibco**	**HU1456**	**Female**	**57**	**23**	***GT***
	**HU1882**	**Male**	**49**	**33**	***TT***
	**HU8116**	**Female**	**23**	**23**	***GG***
	**HU8132**	**Female**	**57**	**29**	***GT***
	**HU8205**	**Male**	**57**	**28**	***GG***
	**HU8230**	**Female**	**47**	**27**	***GG***
	**HU8232**	**Female**	**31**	**18**	***GG***
	**HU8235**	**Male**	**64**	**27**	***GG***
	**HU8251**	**Female**	**56**	**26**	***GG***
	**HU8257**	**Male**	**56**	**24**	***TT***
	**HU8276**	**Male**	**55**	**24**	***GT***
	**HU8289**	**Male**	**22**	**23**	***GG***
**Lonza**	**HUM180851**	**Male**	**47**	**29**	***GT***
	**HUM4242**	**Male**	**40**	**21**	***GG***

Donor demographics (n = 14) and genotype at *rs174537* are provided. A total of 8 *GG*s, 4 *GT*s and 2 *TT*s were obtained. All donors were Caucasian. PHHs obtained from Gibco were provided by the Wake Forest Comprehensive Cancer Center and the two commercial vials from Lonza were purchased. Despite the limited size of this cohort, the genotypic distribution of *rs174537* in the donor samples (57% *GG*, 28% *GT*, and 14% *TT*) is representative of the overall allele frequency observed for Caucasian populations [15,20].

### 3D hepatic organoid cultures

The primary human hepatocytes (PHHs) were seeded at 20x10^6^ cells/ml (~200,000 cells/organoid) in a modified hyaluronic acid (HA)/gelatin-based hydrogel with a polyethylene glycol diacrylate (PEGDA) crosslinker (HyStem^®^-HP, ESI-BIO, Alameda, CA) as previously described. [21–23] This hydrogel was modified to include thiolated fibronectin to promote improved cell adherence and function. Briefly, thiol-modified HA with heparin (Heprasil) was dissolved in sterile water containing 0.5% w/v of the photo initiator 2-hydroxy-4’-(2-hydroxyethoxy)-2-methylpropiophenone (Sigma, St. Louis, MO), creating a 1% w/v solution. Thiolated gelatin (Gelin-S) and the PEGDA crosslinker were dissolved in 0.5% w/v of the same photoinitiated, creating a 2% w/v solution. Millipore^™^ Chemicon^™^ Human Plasma Fibronectin (Millipore Sigma, Cat#FC01010mg, Burlington, MA) was thiolated using a Novus Biologicals^™^ thiolation kit and protocol (Cat#419–0002, Littleton, CO), such that the resultant solution was 1mg/ml. Heprasil, Gelin-S, Extralink, and the thiolated fibronectin were then mixed in a 2:1:1:1 ratio by volume, respectively. Solutions formed a transparent hydrogel when mixed together and was used to resuspend PHHs (20x10^6^ cells/ml). After resuspension of the PHHs in the hydrogel, ultra-violet light (UVGL-58 Handheld UV Lamp, 254 nm, 6 W, UVP, Upland, CA) was used for 15 seconds to initiate the near instantaneous secondary mechanism which stabilized the construct. 3D Hepatic organ-like constructs, from each individual donor, were constructed in a polydimethylsiloxane (PDMS)-coated 48 well plate, by aliquoting 10 ul per well (~200,000 PHHs/organoid). Due to the lack of matched donor hepatocytes with non-parenchymal cells we were limited to a monoculture of PHHs. Hepatic organ-like constructs were allowed to stabilize at 37°C for 30 minutes before they were submerged in 250 μl Williams-E media and placed in an incubator at 37°C with 5% CO_2_ for up to 7 days.

### Cell viability

Viability was qualitatively examined using LIVE/DEAD^®^ Viability/Cytotoxicity Kit assays (Invitrogen, Cat#L3224, Carlsbad, CA) and quantitatively analyzed using an adenosine triphosphate (ATP) quantification kit as previously described [24]. For LIVE/DEAD analysis, tissue constructs were exposed to 2.0 μM calcein AM and 4.0 μM ethidium homodimer in a solution containing Dulbecco’s phosphate buffered saline (D-PBS):media (1:1 v/v), and allowed to incubate for 20 minutes at 37°C. Imaging of hepatic organ-like constructs was performed using a macro-confocal microscope (Leica TCS LSI, Leica, Wetzlar, Germany). For quantitative analysis, the CellTiter-Glo^®^ Luminescent Cell Viability Assay (Promega, Cat#G7571, Madison, WI) was utilized with a Synergy H1 microplate reader (BioTek Instruments, Inc., Winooski, VT) to quantify ATP content. This assay uses a luminescent signal which is proportional to the amount of ATP present, which in turn is proportional to the number of active hepatocytes, according to the manufacturer’s instructions.

### De novo lipogenesis and lipid esterification quantification with [^3^H]-Oleate and [^14^C]-Acetate

Hepatic constructs were evaluated for de novo lipogenesis and lipid esterification functionalities, using established methods previously described [25,26]. Briefly, 3D hepatic cultures were lipid-starved overnight in Williams E media with lipoprotein-deficient FBS overnight (Kalen Biomedical, 880100–5) prior to feeding experiments. Media was supplemented with 10 μCi/ml [^3^H]-oleate (PerkinElmer #NET289005MC) and 1 μCi/ml [^14^C]-acetate (PerkinElmer#NEC084A001MC) with 100 nM of insulin for 90 minutes. Lipids were extracted from the cultures using 3:2 hexane: isopropanol solvent, and sorted into classes using thin-layer chromatography (TLC). The classes were scraped, and levels of radioactivity quantified with a scintillation counter in order to determine the quantity of [^3^H]-oleate and [^14^C]-acetate processed into each class (i.e., phospholipid, triglyceride, free cholesterol and cholesterol ester). The hepatic constructs were re-hydrated and scraped following lipid extraction, and DNA was extracted using the Qiagen DNA/RNA Extraction Mini Kit. Quantification of DNA was performed on a DeNovix. Scintillation counts were normalized to total DNA from each hepatic construct.

### Cholesterol efflux quantification

Hepatocytes assemble cholesterol into very low density lipoprotein (VLDL) particles for secretion or into nascent high density lipoprotein (HDL) particles by ABCA1 [27]. To determine if hepatocytes cultured in 3D were able to efflux cholesterol, cholesterol accumulation in media was measured as previously described [28,29]. 3D cultures were allowed to incubate in normal media overnight. Hepatic constructs were washed twice with warm PBS, and radiolabeling medium was added consisting of William-E media containing 1% fetal bovine serum (FBS) and 2 μCi/ml ^3^H-cholesterol (Perkin Elmer, Cat#NET139250UC) for 24 hours. After the radiolabeling period, hepatic constructs were washed twice with warm PBS and incubated in medium containing 0.2% (w/v) bovine serum albumin (BSA) overnight. Cholesterol efflux was initiated by adding media containing 0.2% BSA or 2% human plasma (v/v). Four hours later, medium was harvested and a 100 μL aliquot was directly counted using a scintillation counter to determine radiolabel in medium. Hepatic constructs were harvested and dried by placing in a 37°C oven for 30 minutes before lipid extraction using 500 μl of 3:2 hexane:isopropanol overnight. The solvent was removed and evaporated under nitrogen before lipids were resuspended in 500 μl of hexane:isopropanol. A 100 μl aliquot was taken, dried under nitrogen and prepared for scintillation counting with a Beckman Coulter LS6500 system (Brea, CA) to determine radiolabel remaining in cells. Cholesterol efflux was calculated as: [(^3^H DPM in media)/(^3^H DPM in media+^3^H DPM in cell extract)]x100. Scintillation counts (disintegrations per minute, DPM) were normalized to DNA (ng) extracted from the protein extract, following lipid extraction and cellular digestion using 0.1 N NaOH.

To analyze distribution of ^3^H-radiolabel across lipoprotein species after completion of the efflux study, 200 μL of pooled media from each group was applied to a Superose 6 Increase 10/300 GL gel filtration column (GE Healthcare; 29-0915-96), as previously described [30]. The samples were eluted at a flow rate of 0.4 mL/min in standard Tris buffer (10 mM Tris, 0.15 M NaCl, 1mM EDTA, 0.2% NaN_3_) and eluate was collected as one fraction per minute. Each fraction was subsequently transferred to liquid scintillation vials for counting [30].

To determine cholesteryl ester (CE) and free cholesterol (FC) distribution in media at completion of the efflux study, 50 μL of media from each sample were lipid extracted using the Bligh-Dyer method [31]. Lipid extracts were subsequently separated by TLC using Silica Gel 60 plates and a solvent system containing hexane:diethyl ether:acetic acid (80:20:2, v/v/v) [31]. This extract was fractionated into its complex lipids components by TLC separation on Silica Gel H plates (no binder; Analtech, Newark, DE) in a hexane/ether/acetic acid (80:20:1, v/v/v) solvent system. The lipid fractions were visualized under ultraviolet light after treatment of the plate with Primuline spray. Each fraction, namely, phospholipid (PL), diglycerides (DG), free fatty acids (FFA), triglycerides (TG), and cholesterol esters (CE), was scraped from the plate and added to a tube containing an internal standard Lipids were visualized by exposure to iodine vapor, and fraction bands corresponding to free cholesterol and cholesterol esters were scraped and counted using a scintillation counter.

### Induction of LA-enriched media

To tightly control fatty acid content of the media, we used lipoprotein-deficient FBS (Alfa Aesar^™^, Cat#J65182). A complete summary of the fatty acid content of the cell culture media is provided in S1 Table. Cell culture media was supplemented with LA bound to human serum albumin (CSL Behring 44206-251-05; 12.5g human albumin in 50mL buffered diluent, King of Prussia, PA) to simulate the LA diet. LA was physically complexed to human albumin at a molar ratio of 1:5 at 37°C for 30 minutes with vortex mixing every 5 minutes, following a modified protocol based on Belayev et al [32]. The resultant media contained 50 μM LA, whereas the “Control” group contained equal volumes of human serum albumin alone, to serve as a vehicle control. Hepatic constructs from each donor were cultured in both media for up to 3 days. Cultured hepatocytes were replenished with fresh media every 24 hrs.

### Genotype at rs174537

DNA was isolated from the hepatocytes utilizing standard molecular techniques [33]. Briefly, DNA pellets were isolated, washed, and rehydrated in Tris-EDTA buffer. DNA concentration was determined using a NanoDrop 2000/2000c Spectrophotometer. Genotyping was determined at *rs174537* using TaqMan methodology on an ABI 7500 real-time PCR machine. The primers for this assay were 5′ Capture: 5′- ACGTTGGATGAGCACCATGTCTGCTGTGTG-3′; 3′ Capture: 5′- ACGTTGGATGAGCCCTGTCGCCCTGCAGAA-3′; Extend: 5′- ACGTCGCCCTGCAGAAGAGACAG-3′.

### Hepatic functional metrics

The impact of LA feeding on hepatic functionality was examined through a urea secretion assay (Quantichrom Urea Assay Kit, BioAssay Systems, Cat# DIUR-100), similar to other studies [34]. Briefly, 50 μl of spent media was exposed to a chromogenic reagent, forming a colored complex with urea. The intensity of the color was directly proportional to the urea concentration of the samples. Samples (50 μl) were analyzed in duplicate and intensity measured at 430 nm using a Synergy H1 microplate reader and a standard curve. Urea concentration of the sample was calculated as [(OD_sample_)-(OD_blank_)]/(OD_standard_)-(OD_blank_)x5. We did not quantify albumin production since the media used in this study were supplemented with LA-conjugated to human serum albumin or human serum albumin as a vehicle control.

We also extracted RNA to evaluate CYP expression as a metric of hepatic function. Hepatic constructs were harvested at baseline, prior to diet introduction, and 24 hours post-diet introduction for the extraction of RNA. Hepatic constructs were placed in 10x volume of RNAlater solution (Qiagen, Cat#76104, Germantown, MD). Constructs were pooled in order to obtain sufficient RNA for qPCR analysis. A homogenizer (Bio-Gen PRO200, PROScientific, Oxford, CT) was used to lyse cells before RNA extraction using the RNeasy kit (Qiagen, Cat#74104) according to the manufacturer’s instructions. RNA was extracted and stored at -80°C. For gene expression analysis, RNA was reverse transcribed into cDNA using qScript Supermix (Quanta Bio, VWR, Cat#95048–100, Radnor, PA). Gene expression of *CYP1A1*, a member of the cytochrome P450 family, was used as a metric of hepatic function. *RPS18* was used for normalization, and the 2^-⊗⊗Ct^ method was applied using baseline expression. qPCR was performed on the ABI 7500 real-time PCR machine. Primers were purchased using the following ID from ThermoFisher: Hs02382618_s1 and Hs01375212_m1, respectively and was amplified using TaqMan Gene Expression MasterMix (Cat#4369016).

### Histology and immunohistochemistry (IHC) staining

Hepatic constructs were harvested for histological characterization over 7 days following initial hepatocyte seeding. Cultured hepatic constructs were washed twice with D-PBS before immersion in 4% paraformaldehyde for 2 hours, and then were rinsed twice with D-PBS, and stored in 70% ethanol. Hepatic constructs were then dehydrated with graded ethanol washes followed by xylene before embedding in paraffin. Sections (5 μm) were created from paraffin-embedded constructs and deparaffinized for staining. IHC was performed after antigen retrieval to examine levels of connexin-32 (Cx32, Invitrogen Cat#14-9759-82: Mouse IgG1 monoclonal antibody, 1:100), organic solute transporter-alpha (OST-α, Invitrogen Cat#PA5-58664: Rabbit polyclonal antibody, 1:50), and phalloidin (Invitrogen, Cat#A22287, 1:50). Cx32 is the major connexin protein found in adult human hepatocyte to hepatocyte junctions [35,36]. OST-α plays a central role in the transport of bile acids, and phalloidin marks the actin cytoskeletal lining of the hepatocytes. Samples were viewed at 40X magnification (Leica DMi8). Additionally, hematoxylin and eosin (H&E) stains were obtained to determine morphology in the 3D hepatic constructs. 40X H&E images were obtained using an Olympus BX63 motorized upright microscope (Shinjuku, Tokyo, Japan).

### Lipid extraction from media and quantification of fatty acid methyl esters

The fatty acid profile, including evaluation of complex lipids, was assessed in 3D hepatic constructs cultured in both control and LA-enriched media. Briefly, 25 μl of media was subjected to a total lipid extraction using an acidified Bligh-Dyer method (10 μg of triheptadecanoin, NuChek Prep, Elysian, MN), subjected to base hydrolysis, and the resultant fatty acids were derivatized to methyl esters (FAME). FAME were quantified using gas chromatography with flame ionization detection (GC-FID) and identified using a Hewlett Packard 7890 instrument system with an Agilent J&W DB-23 column (30 m, 0.25 mm ID, 0.25 μm film) fitted with an inert pre-column (1 m, 0.53 mm ID) for cool-on column injection as previously described [37].

### Intracellular fatty acid quantification

Lipids were extracted from each hepatic construct following exposure to LA and HSA vehicle control using 500 μl of 3:2 hexane:isopropanol overnight. The solvent was removed and evaporated under nitrogen before lipids were converted to FAME and quantified using GC-FID, as previously described [37,38]. Samples were analyzed by GC-FID on a HP 7890 (Agilent Technologies, Inc., Santa Clara, CA) with a DB-23 column as described above using triheptadecanoin (100μg; NuChek Prep) as an internal standard. Fatty acids were cleaved from complex lipids and converted to methyl esters in duplicate samples when possible, utilizing a modification of the protocol developed by Metcalfe et al [39] and Sergeant et al [37] Fatty acids in samples were identified based on retention times of commercially available internal standards. Approximately 12–25 peaks were identified and accounted for >99% of the total fatty acids in the sample. Fatty acid data are presented as the total mass in sample (μg) normalized to protein content (mg).

### Total protein quantification

Total protein was quantified using the Pierce BCA Protein Assay (Cat#23225). Fatty acids were extracted from the hepatic constructs, then cellular residue was dissolved using 0.1N NaOH for 48 hours. The resulting solution was stored at -20°C until further analysis using the Pierce BCA Protein Assay. Standard manufacturer’s protocols were used and each sample was analyzed in duplicate following by a 30-minute incubation at 37°C, absorbance and read at 562 nm. Protein concentrations were determined using a Synergy H1 microplate reader (BioTek Instruments, Inc.) and a standard curve.

### Determination of FADS1 and FADS2 expression

As previously described, hepatic constructs were harvested at baseline, prior to diet introduction, and 30 minutes, 4 hours and 24 hours post-diet introduction for the extraction of RNA. Gene expression of *FADS1* and *FADS2* were determined at 0, 0.5, 4, and 24 hours post-feed in constructs supplemented with LA or HSA control. PCR analysis was conducted in duplicate and normalized to endogenous reference *RPS18* or TBP and relative to a calibrator (2^-⊗⊗Ct^). Primers were purchased using the following ID from ThermoFisher: Hs01096545_m1, Hs00927433_m1, Hs01375212_m1, respectively and was amplified using TaqMan Gene Expression MasterMix (Cat#4369016). Expression analysis was performed using an ABI 7500 real-time PCR machine.

### Statistical analysis

Primary human hepatocytes were obtained from a total of 14 individual donors as illustrated in Table 1. All assays were performed in duplicate when possible. Lipid species differences between diet medium as identified by TLC were analyzed using t-tests and the Satterthwaite method of approximation. Repeated measures analysis of variance (ANOVA) was used to determine changes over time. Box-Cox power transformations (e.g., natural logarithm) were used to transform variables as needed to meet distributional assumptions. To take into account levels of DGLA that were undetectable, the ARA/DGLA ratio was calculated by adding “1” to each fatty acid, such that the ARA/DGLA = [ARA+1]/[DGLA+1]. Cellular fatty acid levels were tested for associations with genotype at *rs174537* using a linear regression model adjusted for diet and time, using time = 0 as the referent point. Gene expression levels were normalized using the ddCt method with GG as the reference gene, and analyzed using a linear regression model with post-hoc Tukey tests. The data presented are mean ± standard error of the mean, unless otherwise noted. All statistical analyses were conducted using SAS (v9.4, Cary, NC) with a significance level of 0.05. No adjustments for multiple comparisons were made.

## Results

### Characterization of 3D hepatic constructs for the study of fatty acid and lipid metabolism

**Fig 1:**
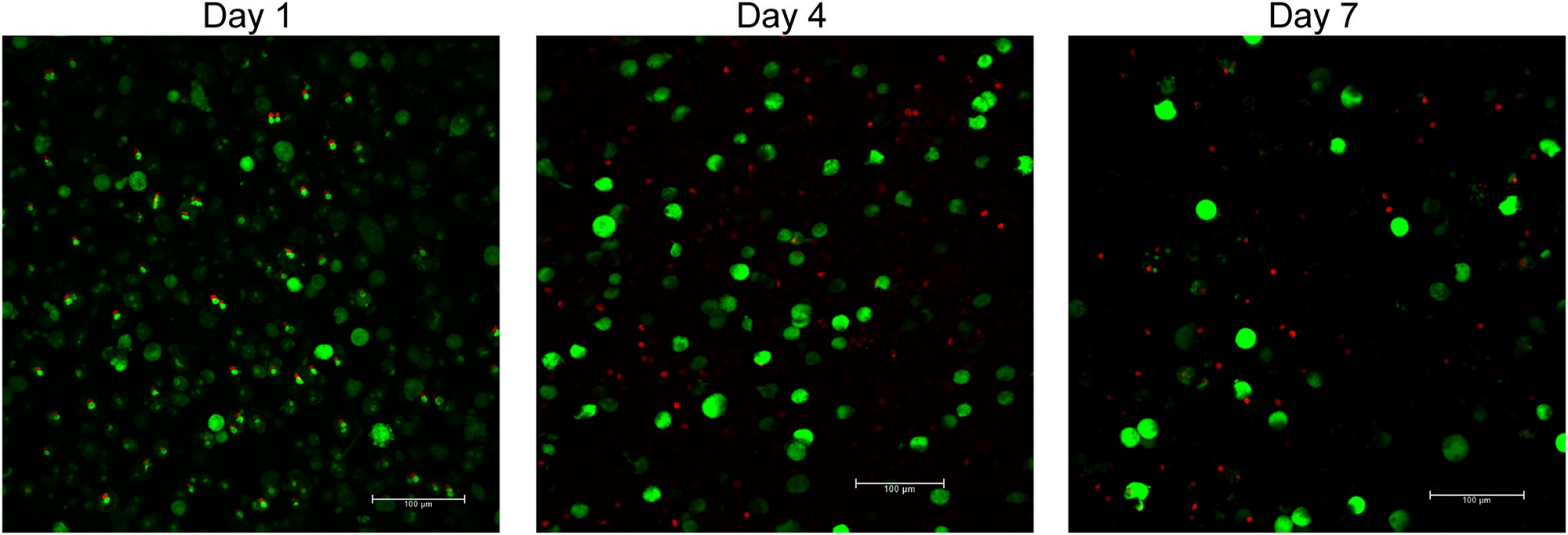
Viability of 3D human hepatic cultures. Imaging of 3D hepatic constructs was performed using a macro-confocal microscope at 20X magnification. Representative LIVE/DEAD images are shown at days 1, 4, and 7. Live cells are marked as green and dead cells are marked by red nuclei. Large clusters/aggregates of viable hepatocytes are observed. All hepatic constructs remained viable for at least seven days of culture. All scale bars are 100 μm.

PHHs were cultured in a custom hydrogel, and were viable for up to one week in culture (Fig 1), and maintained their rounded phenotype for longer than those cultured in 2D (S1 Fig). The constructs also stained positive for connexin-32 and actin filaments (S2 Fig). The entire hepatic construct embedded in the hydrogels were approximately 500 μm in diameter.

**Fig 2:**
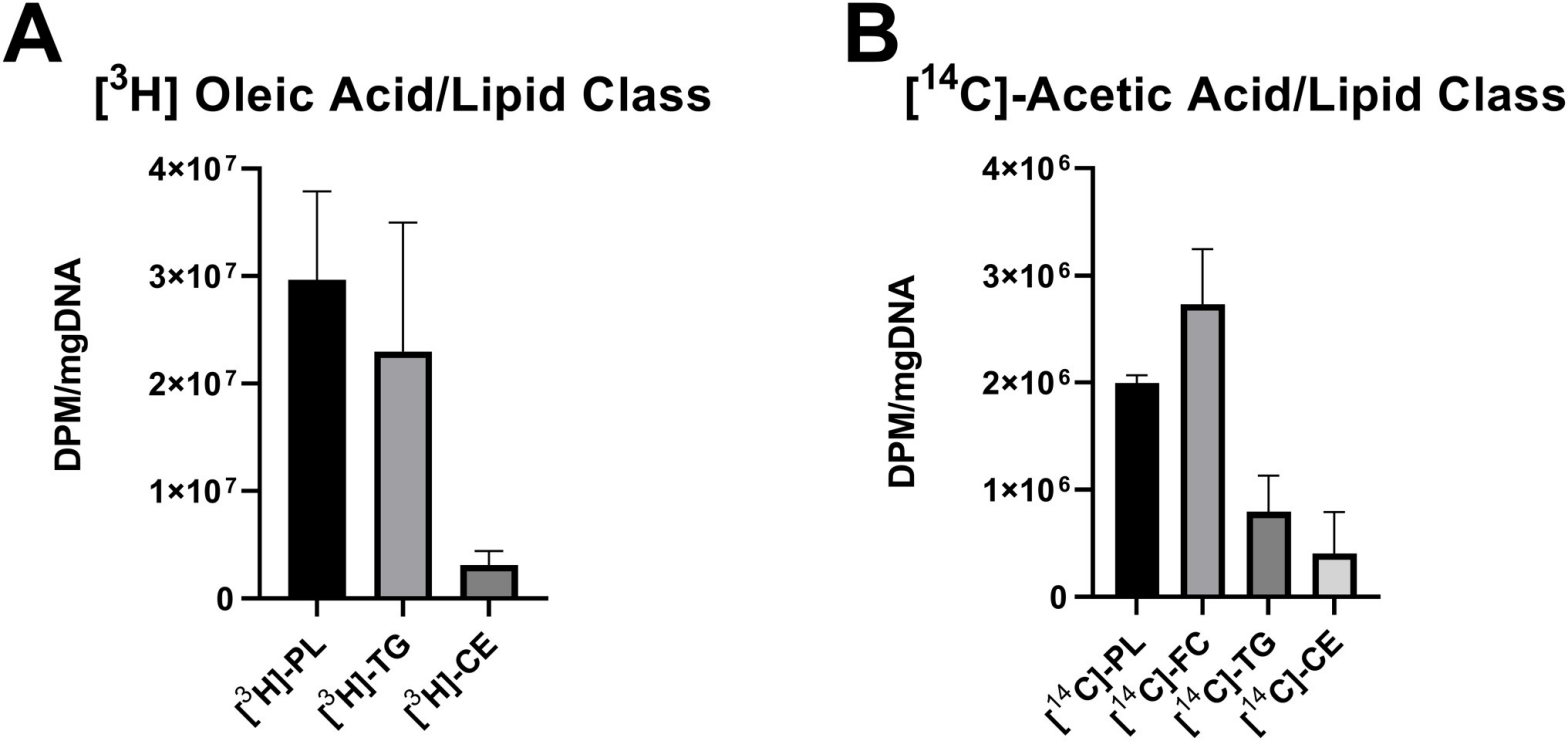
Lipid metabolic functionality in 3D human hepatic constructs. The hepatic construct was incubated with [^3^H]-oleic acid and [^14^C]-Acetic acid for 1.5 hours (n = 3) to quantify lipid esterification and lipogenesis functionality, respectively. (A) Formation of [^3^H]-phospholipid (PL), [^3^H]-triglyceride (TG), and [^3^H]-cholesteryl ester (CE) from of [^3^H]-oleic acid was measured to access the ability of fatty acid esterification. (B) De novo synthesis of [^14^C]-PL, [^14^C]-free cholesterol (FC), [^14^C]-TG, and [^14^C]-CE from [^14^C]-acetic acid were measured fractions. Scintillation counts were normalized to total DNA, and mean ± SEM are illustrated. DPM, disintegrations per minute.

The liver is a metabolically active organ; it is responsible for converting dietary fatty acids and repackaging them into different forms before they are transported to other tissues and organs within the body [40]. In order to evaluate the capacity of the cultured hepatocytes in 3D to represent these key lipid metabolic function, the hepatic constructs were fed with [^3^H]-oleic acid for 1.5 hours. Lipids were separated into phospholipid, free cholesterol, triglyceride and cholesterol ester fractions and the amount of [^3^H]-oleic acid present in each class was quantified. We observed that 3D human hepatic constructs were able to incorporate [^3^H]-oleic acid into other lipid species (i.e., fatty acid esterification). Successful conversion into phospholipids (29.89±4.6% of detected downstream lipid species), cholesterol esters (3.86±0.38%), and triglycerides (32.28±7.2%) was observed (Fig 2A). We then used [^14^C]-acetic acid to quantify functional *de novo* lipogenesis in the 3D hepatic constructs. Similar to before, hepatic constructs were exposed to [^14^C]-acetic acid for 1.5 hours. Newly synthesized phospholipids (32.01±2.3% of detected lipid species), free cholesterol (42.82±5.1%), triglyceride (17.99±5.1%), and cholesterol esters (7.18±2.5%) from [^14^C]-acetic acid were observed (Fig 2B). The successful conversion of these radiolabeled fatty acids to more complex lipid species indicates that the 3D hepatic culture platform is capable of replicating *in vivo* lipid metabolic function.

### Normal rates of cholesterol efflux observed in 3D hepatic constructs

**Fig 3:**
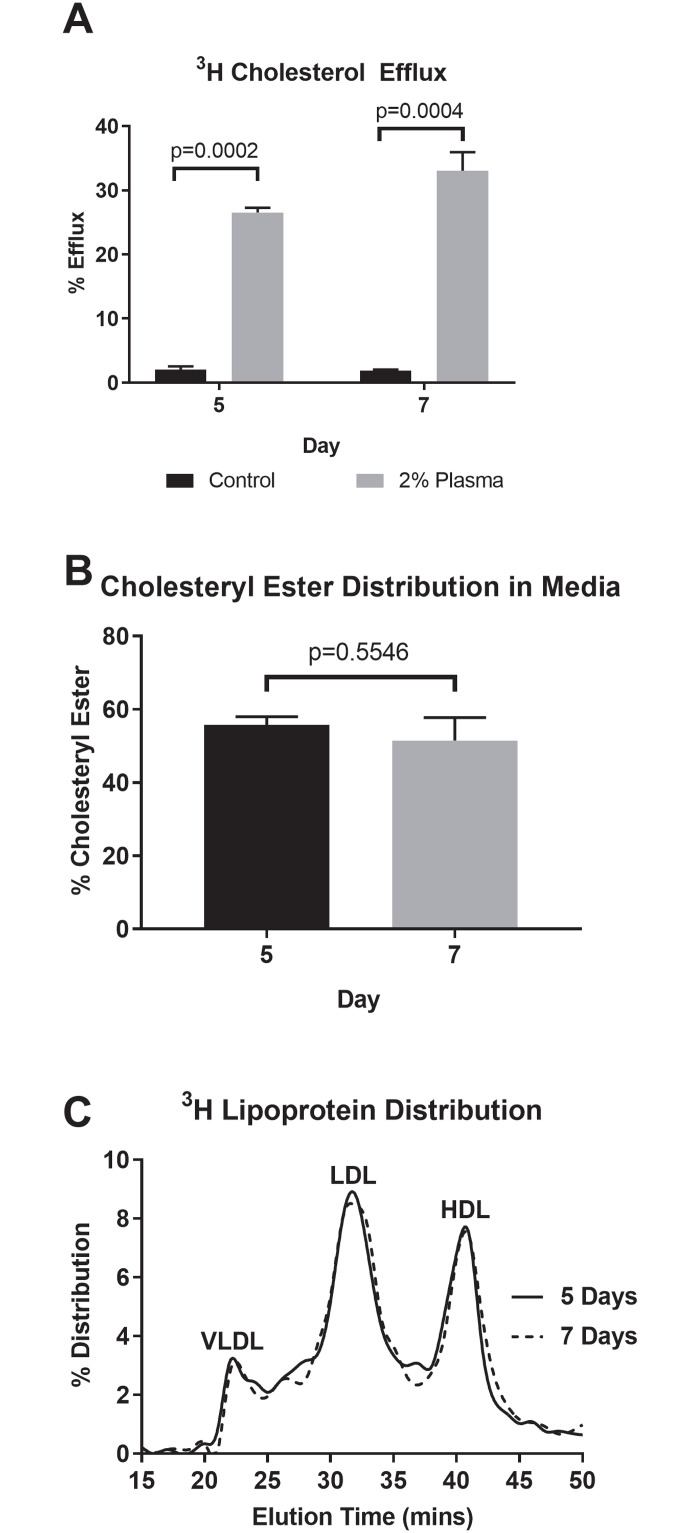
Cholesterol efflux from 3D cultured hepatic constructs. Cholesterol efflux supported by lipoproteins is a key property of hepatocytes. To assess efflux, PHH were cultured in 3D and radiolabeled with ^3^H-cholesterol for 24 hours after 5 and 7 days post culture. (A) Hepatocytes were subsequently washed and incubated with 2% human plasma or 2% BSA to initiate efflux over a 4 hour time frame. Incubating hepatocytes with media containing 2% plasma resulted in significant radiolabeled cholesterol efflux compared to cultures incubated with 2% BSA alone. Mean ± standard error of the mean is reported. There were no significant differences in cholesterol efflux between hepatic constructs cultured at days 5 and 7. (B) The proportion of radiolabeled cholesteryl ester in media was quantified, revealing more than 51% of the cholesterol efflux from the cultures was recovered as cholesteryl ester on days 5 and 7. (C) Media from hepatocytes incubated with human plasma was fraction by fast protein liquid chromatography to determine ^3^H-radiolabel lipoprotein distribution. The distribution of hepatocyte conditioned media ^3^H-cholesterol among very low-density lipoprotein (VLDL), low-density lipoprotein (LDL), and high-density lipoprotein (HDL) was similar between days 5 and 7 (n = 3 per group).

Cholesterol efflux refers to the movement of cholesterol out of hepatocytes in the form of low-density lipoprotein (LDL) and high-density lipoprotein (HDL) particles. An increase in LDL production has been shown to contribute to inflammatory diseases, such as atherosclerosis, whereas HDL promotes efflux of cellular cholesterol and therefore contributes to the attenuation of inflammation [41]. In order to demonstrate the feasibility of using 3D human hepatic constructs for the study of lipid metabolism, their capacity to release cholesterol in response to stimuli and produce lipoproteins was quantified. Hepatic cultures were radiolabeled with ^3^H-cholesterol before stimulating its release with 2% plasma. We observed 3D hepatic constructs at 5 days of culture efflux significantly more radiolabeled cholesterol in the presence of 2% plasma compared to 2% bovine serum albumin (BSA) (26.6% vs. 2.03%, respectively, p = 0.0002, Fig 3A). This trend continued with hepatic constructs harvested on day 7. As expected, we observed significantly more radiolabeled cholesterol efflux from hepatic constructs stimulated with plasma compared to those stimulated with BSA (33.0% vs. 1.87%, respectively, p = 0.0004). Lipids were extracted and separated using thin layer chromatography (TLC). Of the secreted cholesterol, we observed that 56% was cholesteryl ester on day 5 and 51% on day 7 (Fig 3B). This indicates that a significant portion of the cholesterol that was in the media was either being secreted in VLDL particles as CE, or was efflux onto HDL particles as free cholesterol with subsequent esterification to CE by lecithin-cholesterol acyltransferase, and not likely diffusion from the cells into the media. Importantly, conditioned media ^3^H-cholesterol distribution among lipoproteins was similar between days 5 and 7 (Fig 3C). Taken together, these results support the successful efflux of cholesterol by the 3D cultured hepatocytes and cholesterol secretion was maintained over a period of one week.

### Hepatocyte fatty acid composition changes upon LA exposure

**Fig 4:**
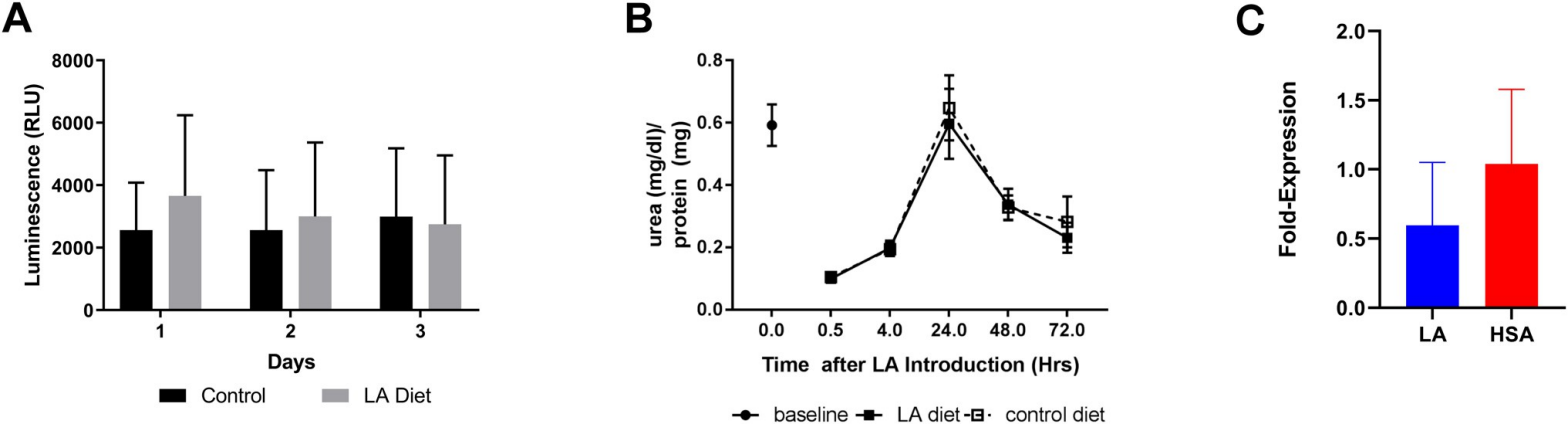
Hepatocyte viability is maintained over study period. (A)There was no significant difference in cell viability between LA and control groups at any time based on luminescence measurements from the ATP quantification assay (n = 3/group). (B) Hepatic functionality, as measured by urea, was not impacted by LA feeding over 3 days of culture. Maximum urea levels were detected 24 hours post-feeding in both LA and HSA-control groups, indicating that the constructs were capable of secreting urea up to baseline levels following LA or HSA exposure. (C) The expression levels of CYP1A1, a member of the cytochrome P450 family, were not significantly impacted by feeding with LA or HSA when compared to pre-feeding levels.

As the 3D human hepatic cultures were demonstrated to be capable of lipogenesis, lipid esterification and cholesterol efflux during our initial characterization, we then conducted a pilot study to evaluate the feasibility of assessing acute SNP-diet interactions, with respect to SNP *rs174537* and essential dietary omega-6 PUFA: LA, within the 3D hepatic culture platform. Hepatic constructs were exposed to LA-enriched and control fatty acid media (S1 Table) for up to 3 days, consistent with the best viability and hepatic metabolic functionality previously observed. PHHs from individual donors were incubated with or without 50 μM of human serum albumin (HSA)-conjugated LA. Viability of the constructs was not significantly affected by LA incubation compared to HSA vehicle controls and was stable over the three day study period, as indicated by LIVE/DEAD staining and adenosine triphosphate (ATP) quantification (Fig 4). Cell viability visualized via the LIVE/DEAD stain and our ATP quantifications correlated well and we did not detect high levels of apoptosis or necrosis. Urea secretion into the media was used to quantify hepatic construct functionality over time. As anticipated, urea secretion reached baseline levels (0.62 ± 0.34 mg/dl normalized to mg of total protein) within 24 hours media depletion and LA exposure [42]. There was a drop in urea at 48 and 72 hours but there were no significant differences in urea concentrations between the control and LA groups over the course of this study. Given these findings, we performed the remainder of the experiments with hepatic constructs within 24 hours of culture. The expression of CYP1A1 was not significantly impacted by feeding with LA or HSA (0.60 ± 0.19 and 1.04 ± 0.204 fold changes in expression compared to baseline, respectively) (Fig 4C).

**Fig 5:**
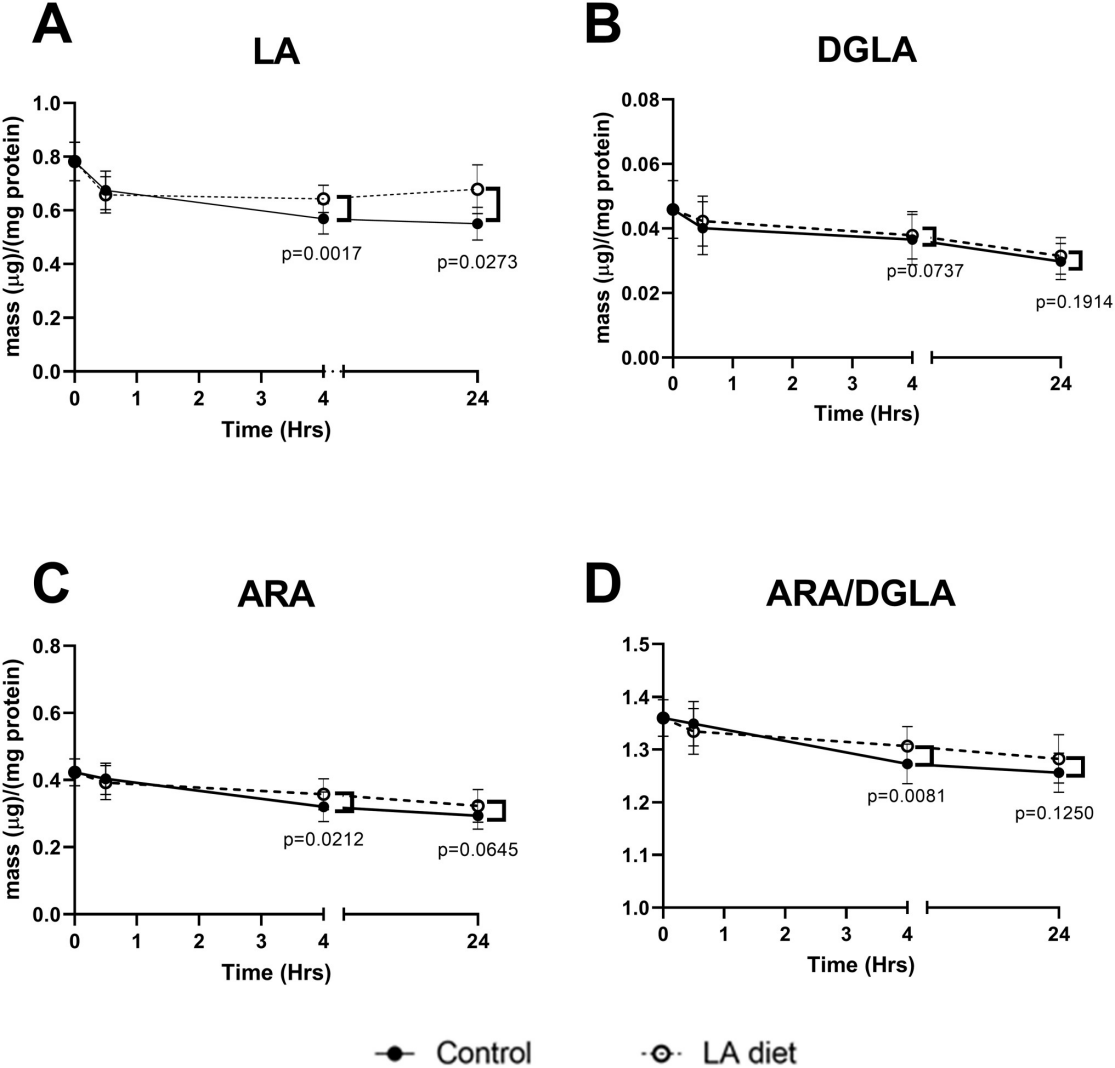
Cellular fatty acid content after incubation with LA. Cellular lipids were extracted from 3D hepatic constructs and analyzed as fatty acid methyl esters (FAME). Mean and standard error of the mean (SEM) are reported. Differences between LA (dotted lines) and control (solid line) groups at each time point were analyzed using paired t-tests. N = 12 per group. (A) Hepatocyte LA levels were significantly higher at 4 hours (p = 0.002) and 24 hours (p = 0.03) post-LA diet exposure. (B) DGLA levels did not differ significantly over time or between diet groups (p = 0.27, p = 0.82, respectively). (C) ARA levels were significantly higher at 4 hours after LA diet exposure (p = 0.02). (D) ARA/DGLA ratio was significantly higher in constructs exposed to LA diets at 4 hours (p = 0.008).

Due to the rapid metabolism of fatty acids by the 3D hepatic constructs, the remainder of the analysis is focused on the first 24 hours of culture after diet exposure. Hepatic constructs and media aliquots were harvested at 30 minutes, 4 hours, and 24 hours post-diet exposure. Cellular fatty acid content (i.e., from hepatocytes) was quantified as fatty acid methyl esters (FAME) by gas chromatography with flame ionization detection (GC-FID). We observed trends towards an increase in saturated fatty acid levels (i.e., palmitic, stearic, and oleic acids) within 24 hours in all hepatic constructs, regardless of diet (S2 Fig). Importantly, we began to detect an increase in cellular levels of LA at 4 hours, which was sustained at 24 hours post-LA diet exposure (Fig 5A). Investigating the downstream products of LA metabolism, dihomo-gamma-linolenic acid (DGLA) levels were relatively consistent over time were not significantly different between diet groups (Fig 5B). However, ARA levels were significantly higher at 4 hours (p = 0.0212) post LA-diet exposure (Fig 5C). As a result, the ARA/DGLA ratio, which is a surrogate measure of FADS1 activity, were significantly higher in the LA group at 4 hours, compared to controls. Please note that ARA/DGLA ratios presented in Fig 5D. have been mathematically transformed, as explained in the methods section. One noticeable trend we observed consistently across all hepatic constructs, regardless of diet group, was that cellular levels of n-6 PUFAs gradually decreased over time, albeit not always significantly. We attribute these changes to natural variability in functionality of PHHs and slight changes in viability over time.

### Hepatic constructs demonstrate allele-specific metabolic activity

Given that PUFA metabolism in humans is dependent on FADS related genetic variants, we aimed to explore the ability of the 3D human culture platform to replicate individual variability in PUFA metabolic conversion capacity. To demonstrate the allele-specific metabolic activity, we first focused on SNP *rs174537*, a known genetic variant contributing to differential FADS1 activity. We successfully extracted approximately 150 ng of RNA and 750 ng of DNA from each hepatic construct (~200,000 cells). To increase our yield, we pooled 2 constructs as needed to ensure sufficient RNA and DNA yield.

**Fig 6:**
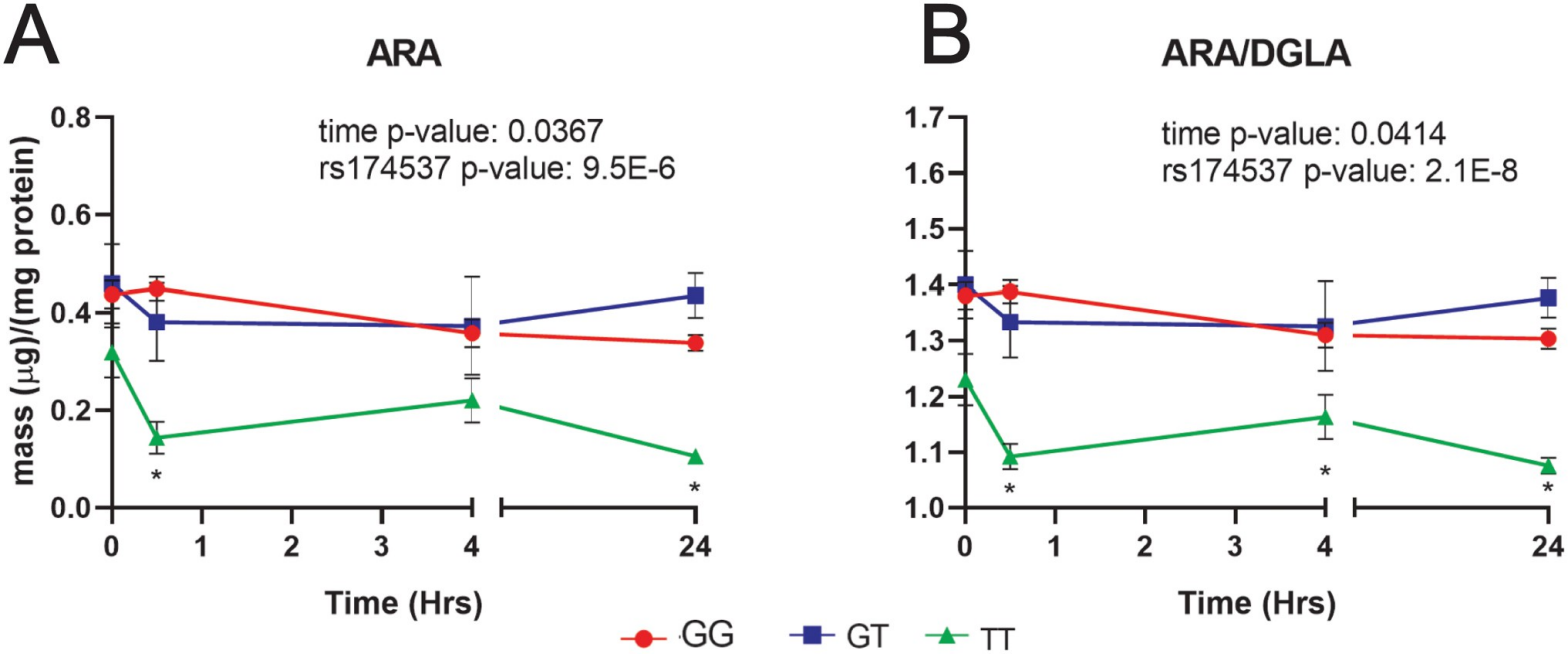
Genotype at *rs174537* is strongly associated with cellular ARA levels and ARA/DGLA ratio in 3D cultured hepatocytes. Effect of genotype at *rs174537* on cellular n-6 PUFAs over time was assess using a linear regression model. Mean ± SEM are displayed. Red circles denote *GG* genotype (n = 14), blue squares signify the *GT* (n = 6), and green triangles mark *TT* genotypes at *rs174537* (n = 4). A) *TT* individuals had lower levels of cellular ARA compared to the *G*-allele counterparts at 30 minutes and 24 hours regardless of diet exposure (p = 0.003, p = 0.0002, respectively), which translated to significant differences in ARA/DGLA ratio (B), Individuals with the genotype *TT* at rs174537 had significantly lower ARA/DGLA levels at all time points (p = 0.0009, p = 0.0247, p = 8.5E-5, respectively). No statistically significant SNP-diet interactions were observed, potentially due to the relatively low dose of LA in the media.

Given previous reports illustrating the strong effect of *rs174537* on ARA and the ARA/DGLA within liver tissues [43], the effect of genotype on the intracellular long chain PUFA composition was investigated. We did not observe significantly differences in the cellular levels of ARA between organ-constructs exposed to LA-diets vs. controls within each genotype (see S4 Fig). However, consistent with previous studies using hepatocytes and whole blood samples, we observed that hepatocyte levels of ARA and the ARA/DGLA varied in association with SNP *rs174537* (p = 9.5E-6 and p = 2.1E-8, respectively, Fig 6A and 6B) regardless of diet group. Individuals who were homozygous with the minor allele (i.e., *TT* at *rs174537*) displayed significantly lower levels of ARA at 30 minutes (p = 0.0033) and 24 hours (p = 0.0002) (Fig 6A). These trends were also replicated in the ARA/DGLA ratio (Fig 6B). These individuals also had significantly lower ARA/DGLA ratios compared to individuals carrying the major allele (i.e., *GG* and *GT*) at 0.5, 4, and 24 hours regardless of the diet group (p = 0.0009, p = 0.0247, p = 8.5E-5, respectively),. These findings are consistent with current literature supporting the most efficient FADS1 dependent conversion of DGLA to ARA for individuals carrying the *G* allele in comparison to those homozygous with minor allele (i.e., *TT*) [15,16,44,45]. Please note that ARA/DGLA ratios presented in Fig 6 have been mathematically transformed, as explained in the methods section.

Next, we examined the SNP-diet effects at times 4 hours and 24 hours with respect to dietary LA exposure, since cellular levels of LA and ARA were significantly different between groups at those times (Figs 5 and 6). Essentially, we observed strong genetic effects (i.e., SNP) in both control and LA groups, but not statistically significant SNP-diet interactions on the cellular ARA levels or ARA/DGLA ratios, possibly due to the relatively low dose of LA used in the media.

### FADS1 expression is associated with rs174537

**Fig 7:**
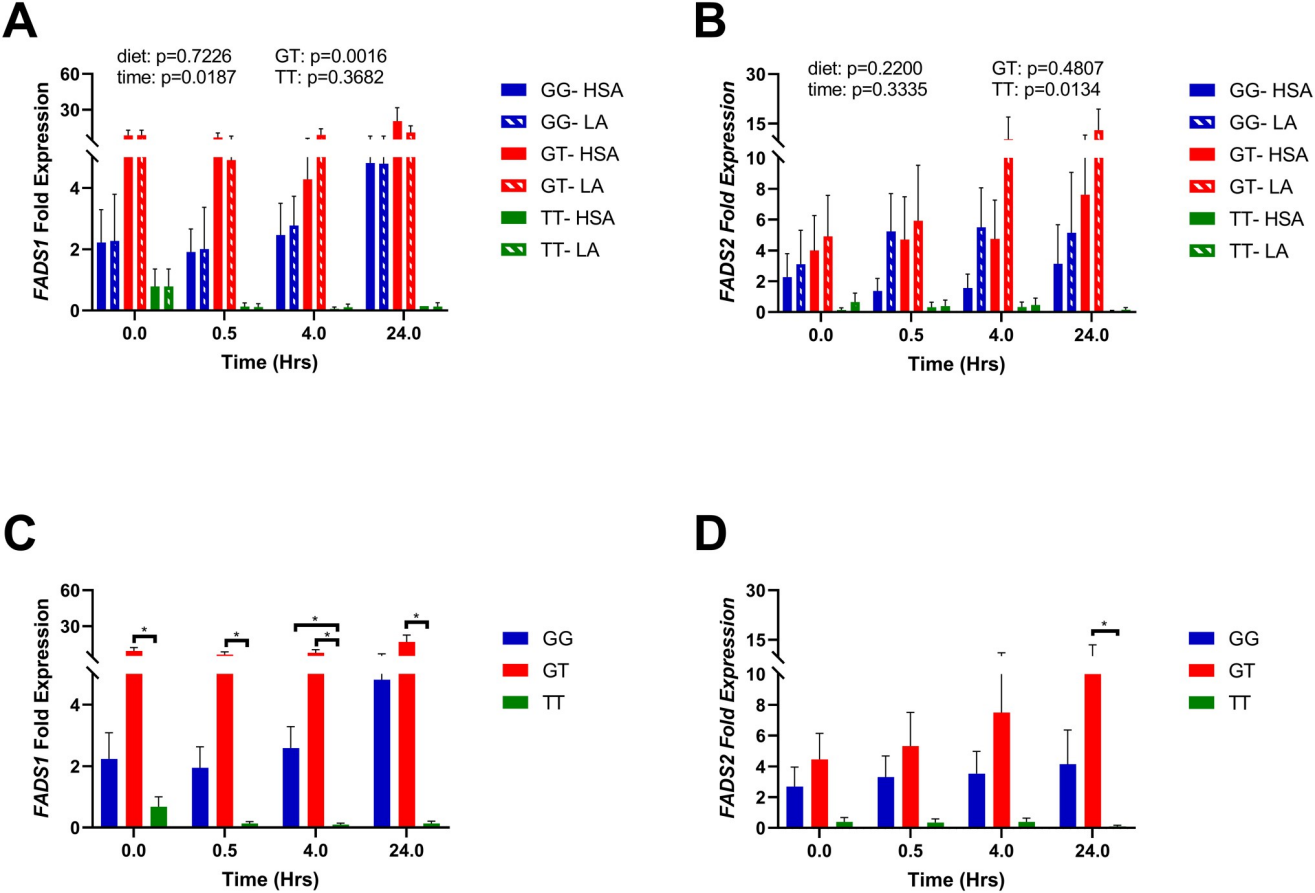
Expression of *FADS1* and *FADS2* is associated with genotype at *rs174537*. Effect of diet and genotype at *rs174537* were analyzed using a linear regression model, where *GG* at *rs174537* was considered the referent genotype. Data are reported as mean with SEM. No significant differences were observed in the expression of (A) FADS1 or (B) FADS2 between LA-exposed and human serum albumin (HSA)-control hepatic culture models. When analyzed by genotype alone, (C) FADS1 expression was significantly higher in GT constructs, and lowest in *TT* constructs. (D) A similar trend was seen in FADS2 expression. Interestingly FADS1 and FADS2 expression levels were highest in *GT* donors.

The expression levels of *FADS1* and *FADS2* were quantified using RT-qPCR and normalized to the *GG* expression levels at time zero using the ddCt method. We used the major allele (i.e., *GG* at *rs174537*) as the reference point for ddCt normalization at time = 0, and *GG* also served as the reference genotype for the linear regression model. FADS1 and FADS2 expression levels were not significantly different between the HSA-control and LA exposed hepatic cultures (p = 0.453 and p = 0.084 respectively) (Fig 7A and 7B). We also tested for SNP-diet interactions, but no statistically significant interaction was detected at any time point. We attribute the lack of a significant SNP-diet interaction to the low dose of LA used in this pilot feasibility study (i.e., 50μM). However, we did detect a genotypic effect. Expression of *FADS1* was significantly higher in hepatic constructs generated from donors who were GT at *rs174537* (p = 0.0016) (Fig 7A). The expression levels of *FADS2* was significantly lower in constructs with genotype *TT* than those with *GG* (p = 0.0134) (Fig 7B).

Given no significant effect between diet groups, we further evaluated the genotypic effect by pooling all the samples and stratifying by genotype only (Fig 7C and 7D). In constructs derived from *TT* donors, FADS1 expression was significantly lower than *GT* levels at all time points, and lower than *GG* hepatic constructs at 4 hours (Fig 7C). Similarly, *FADS2* expression was significantly lower in *TT* cultures than *GT* at 24 hours (Fig 7D). This is congruent with studies in human subjects that have demonstrated lowered downstream PUFA levels in serum samples and liver tissue from *TT* individuals [14,16,17,43].

## Discussion

This study characterized an *in vitro* human liver model of lipid metabolism and genetic variability in PUFA metabolism. We demonstrated that 3D cultured hepatic constructs from individual donors of various age and healthy phenotype can model the physiological conditions of normal lipogenesis, lipid esterification, and cholesterol efflux. 3D Hepatic cultures were then incubated with and without the essential dietary omega-6 PUFA, LA, to assess metabolic conversion of LA to ARA. Cellular levels of LA and ARA increased within 4 hours after dietary exposure. Importantly, the *in vitro* tissue engineered platform captured many *in vivo* phenomena including allele-specific variations in PUFA metabolic conversion capacities and *FADS* expression, which can be instrumental towards gaining new insights into the regulation of fatty acid metabolism and tailoring diets to prevent or treat disease in humans.

Characterization of existing 3D cultured hepatic organoid and spheroid models have predominantly focused on the secretory and drug metabolic functions of the liver, with lipid and fatty acid metabolic function largely ignored. Herein, we characterized an *in vitro* 3D human hepatic culture model for its capacity to form lipids from excess energy, form complex lipid species from dietary free fatty acids, and package cholesterol for transport. Conversion of [^3^H]-oleic acid and [^14^C]-acetic acid into complex lipid species representative of lipid esterification and de novo lipogenesis, respectively were comparable to published reports [25,26]. In addition, cholesterol efflux was successfully demonstrated, even after one week of culture. These results suggest that the 3D tissue engineered model can be further exploited for longer diet exposure studies in the future.

The intake of LA and other omega-6 PUFAs in the MWD has dramatically increased over the past 40 years. Consumption of LA from vegetable oils has increased at least 3-fold in the last decade alone. This dietary shift has contributed to the rising prevalence of diabetes, obesity, cardiovascular disease, and other chronic inflammatory diseases. [1–3,6] Despite large epidemiologic studies revealing the negative impact of high dose LA diets, controversy remains over the optimal amount of LA (or even appropriate) ratios of n-6:n-3 dietary PUFAs for human health. This can be attributed to the growing evidence that PUFA metabolism is not uniform, as once thought, in humans [6,15,16,46]. Notably, SNPs *rs174537* and *rs174550* significantly impact response to LA diets [13] by altering ARA/DGLA in both human liver tissue [43] and blood samples [38,47]. However, studying SNP-diet interactions (e.g., *rs174537* and MWD) in humans by clinical trial is costly and cannot provide direct hepatic measurements of enzyme expression and activity. Therefore, there is an unmet need to define mechanistic pathways relating diet to physiology and that may inform human clinical trials. This study revealed that 3D hepatic constructs enable measuring SNP dependent effects on liver lipid metabolism and enzymatic expression. Individuals homozygous with the minor allele (i.e., *TT*) at *rs174537* had the lowest levels of FADS1 and FADS2 expression, within those exposed to the LA diet, while heterozygous individuals (*GT*) had the highest. This is interesting, since our analysis of the ARA/DGLA ratios were comparable between *GG* and *GT* donors, yet *GT* consistently exhibited the highest FADS1 and FADS2 expression levels. There is a need to further investigate the functional variants responsible for variations in FADS activity. Overall, we successfully replicated the allele-specific variability in PUFA metabolism and FADS1 and FADS2 expression, showcasing the value of this *in vitro* platform to advance future mechanistic gene-diet studies within human liver constructs.

Cultivation of the PHHs in a 3D configuration is an attractive tool since the phenotype and functionality of the hepatocytes is maintained over a longer culture period than can be achieved traditionally in two dimensions (see S1 Fig) [48–50]. Importantly, the inter-individual variation between human donors recapitulates in the 3D tissue engineered culture system. We cultured PHH monocultures, without the presence of supportive non-parenchymal cell types, in a customized hydrogel. We acknowledge that mono-culture of PHHs in this model represents a highly simplified version of hepatic tissue and function, and thus limits its application. In this study, non-parenchymal primary cells were not included due to limited access to primary cells from matched donors that would preserve the genotype at *rs174537*. However, others have been successful in culturing non-parenchymal cells (e.g., stellate, Kupffer, liver sinusoidal endothelial cells) with PHHs [51]. These multi-cellular organ-like constructs have been shown to be viable for up to 30 days. For example, Kozyra et al. successfully demonstrated the use of hepatic spheroids (void of ECM and predominantly composed of PHH) to induce steatosis and insulin resistance [18]. We believe that future studies will be able to incorporate the non-parenchymal cells, especially Kupffer cells, to gain an improved understanding of how dietary PUFAs impact hepatic function and downstream inflammatory responses.

Despite major advances in the current *in vitro* models of the human liver by culturing PHHs in both spheroid and organoid formats, there are some challenges that need to be addressed before this platform can be exploited for high throughput studies involving genetics, metabolomics, and lipidomics [52–54]. Spheroids are generally an aggregation of cells (~1,000–1,500 cells/spheroid) that are cultured in a hanging drop method sans scaffold material. The lack of ECM and small size of these spheroids have been attractive for high throughput drug screening/drug toxicity studies. Yet for successful -omic quantification, these spheroids are pooled to the upwards of one million cells (i.e., 1,000 spheroids are needed) to perform reliable and robust metabolomics or lipidomic analyses. Unlike spheroids, organoid or organ-like construct platforms generally provide biomechanical support to the cells via the presence of ECM components and are larger in size utilizing 200,000–500,000 cells/organoid, which gives rise to more physiologically accurate micro-environments. While the cell numbers are significantly higher than spheroids, the presence of the ECM can present some challenges in successfully extracting adequate RNA and DNA content. In our current study we were able to successfully extract ~150 ng of RNA and 750 ng of DNA from each hepatic construct (~200,000 cells). Hydrogel systems that demonstrate reversible crosslinking may allow for increased yield [55]. Thus, there is a need for future refinements and optimizations of the 3D tissue engineered platforms to improve scalability and cost-effectiveness of this approach. We believe that further optimizations of this platform can also significantly improve the lipid and PUFA metabolic functional capacity not only for short-term/acute experiments but also longer studies (e.g., 2 and 4 weeks) that can greatly enhance our understanding of dietary exposure on hepatic function [56–58].

In this study, we focused on SNP *rs174537*, but we recognize that there are several other genetic variants within *FADS* and *ELOVL* that may be interesting to study for the purpose of SNP-PUFA diet or gene-PUFA diet interactions. In particular, Pan et al. recently identified *rs174557* as a possible functional SNP for *FADS1* [59], which lies within intron 1 of *FADS1* is functional and lies within a PATZ1 binding site which overlaps with a SP1 site. The common allele in most populations diminishes the binding of PATZ1, increasing the activation of SP1 binding to transcription factor SREBP1c, which then acts to regulate expression of *FADS1*. Like us, they hypothesized increasing levels of LA would lead to increased levels of ARA and downstream proinflammatory eicosanoids. While *rs174537* and *rs174556* are in LD, albeit not perfectly (D’ = 1.0, R^2^ = 0.76), further studies using this 3D organoid platform can be designed to better understand these SNP-diet interactions. Additionally, other SNPs within the *FADS* cluster have been associated with *FADS1* and *FADS2* expression levels. For example, *rs174548* has been suggested to be a potential functional SNP for both *FADS1* and *FADS2*; this SNP also appears to be in LD with *rs175437* (D’ = 0.89, R^2^ = 0.70). [1] *rs174556* had the highest correlation to *FADS1* expression in liver tissue samples in a mixed race cohort (n = 154) [60]. Indeed there are several variants that need to be studied in the future to tease out the mechanistic roles they play in regulating PUFA metabolism, not only for Caucasian donors, but African Americans and mixed races where there a major health disparities and differential PUFA metabolic capacities. In this study we were limited to Caucasian donors, but future studies can procure PHHs from ethnically diverse donors.

In summary, the present study characterized the lipid metabolic functionality of 3D human hepatic cultures. More importantly, it illustrates the feasibility of exploiting 3D hepatic systems for SNP-diet interactions that inform the field of precision nutrition. The cultured hepatic constructs differentiate normal and abnormal lipogenesis and cholesterol efflux, discriminate allele-specific metabolic conversion of dietary PUFAs, and are suitable for informing PUFA metabolism relevant to human health and disease. The promising *in vivo* like features of the 3D human hepatic cultures presented here can enable deeper studies into the molecular mechanisms and pathogenesis of precision nutrition and how PUFAs can be used to augment inflammation and disease.

## Supporting information

S1 Fig Live-dead imaging of primary human hepatocytes cultured in 2D vs. 3D constructs. Hepatocytes cultured in 2D on a layer of collagen began to de-differentiate (i.e., lose their rounded morphology) at around 4 days of culture. Conversely, those that were cultured in the 3D configuration maintained their phenotype for at least 7 days.
(TIF)

S2 Fig Immunohistochemistry of 3D hepatic constructs revealed positive staining of Connexin-32 and actin filaments. Blue: nuclei (DAPI), green: connexin-32 (Cx32), gray: actin (right image). Light gray triangle arrows highlight the presence of Cx32. Scale bar = 100 μm.
(TIF)

S3 Fig Saturated fatty acid levels increased over time in LA and control hepatic constructs. No significant differences in saturated fatty acid levels were observe between LA-exposed and control groups.
(TIF)

S4 Fig Cellular ARA and ARA/DGLA ratio by genotype at rs174537 and LA dietary exposure. Exposure to dietary LA did not result in significantly different differences within each genotype. However, TTs consistently exhibited lower (A) ARA and (B) ARA/DGLA levels.
(TIF)

S1 Table Summary of fatty acid content of cell culture media used in this study. Fatty acid methyl esters (FAME) were extracted and quantified using GC-FID. Means and standard error of the mean are reported of the mass of fatty acid per 200 ul volume of media. Standard media refers to Williams-E media with FBS and contains significant amounts of ARA and other PUFAs. For the purpose of this study Control and LA media were formulated using lipoprotein deficient fetal bovine serum (FBS) to obtain a tighter control over PUFA content.
(DOCX)
