# Supplementary figures and images for: Exploiting three-dimensional human hepatic constructs to investigate the impact of rs174537 on fatty acid metabolism
Source: PLoS One. 2022 Jan 20;17(1):e0262173. doi: 10.1371/journal.pone.0262173 (PMC8775235; doi:10.1371/journal.pone.0262173)

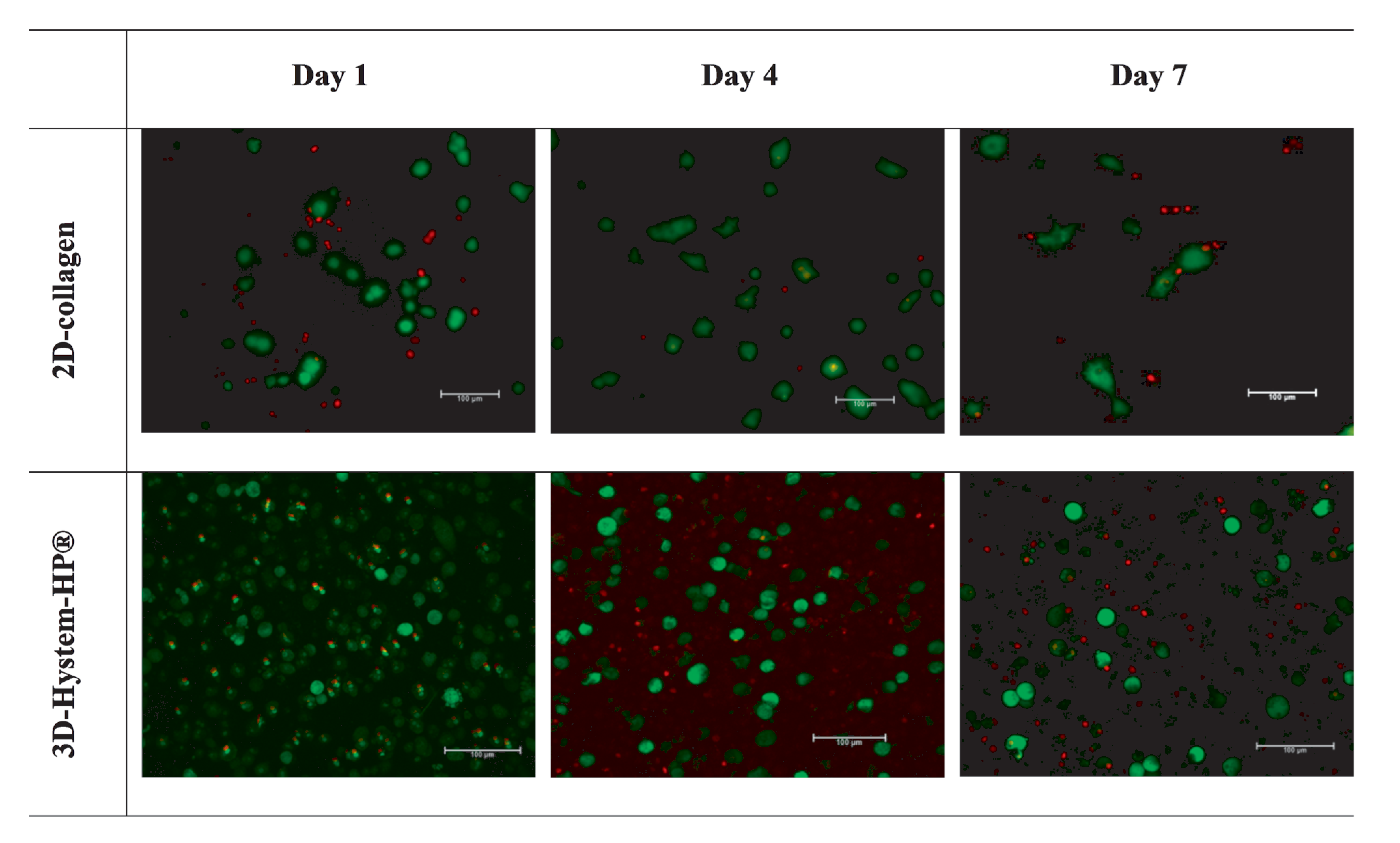

Supplement: S1 Fig — Hepatocytes cultured in 2D on a layer of collagen began to de-differentiate (i.e., lose their rounded morphology) at around 4 days of culture. Conversely, those that were cultured in the 3D configuration maintained their phenotype for at least 7 days. (TIF) [file pone.0262173.s001.tif]

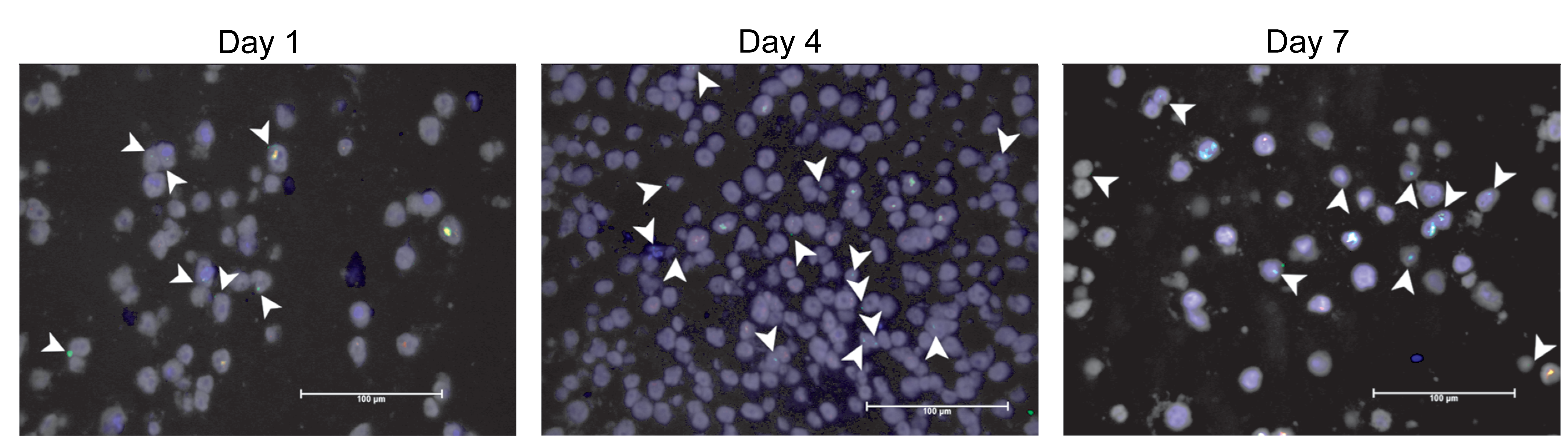

Supplement: S2 Fig — Blue: nuclei (DAPI), green: connexin-32 (Cx32), gray: actin (right image). Light gray triangle arrows highlight the presence of Cx32. Scale bar = 100 μm. (TIF) [file pone.0262173.s002.tif]

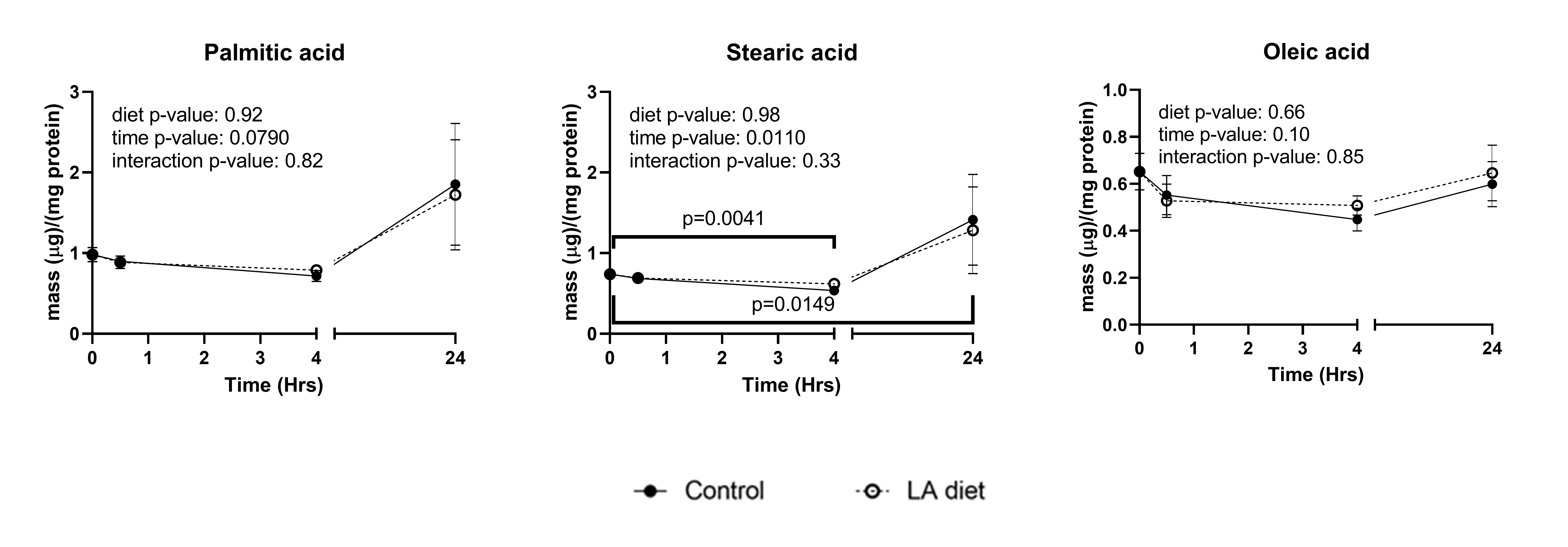

Supplement: S3 Fig — No significant differences in saturated fatty acid levels were observe between LA-exposed and control groups. (TIF) [file pone.0262173.s003.tif]

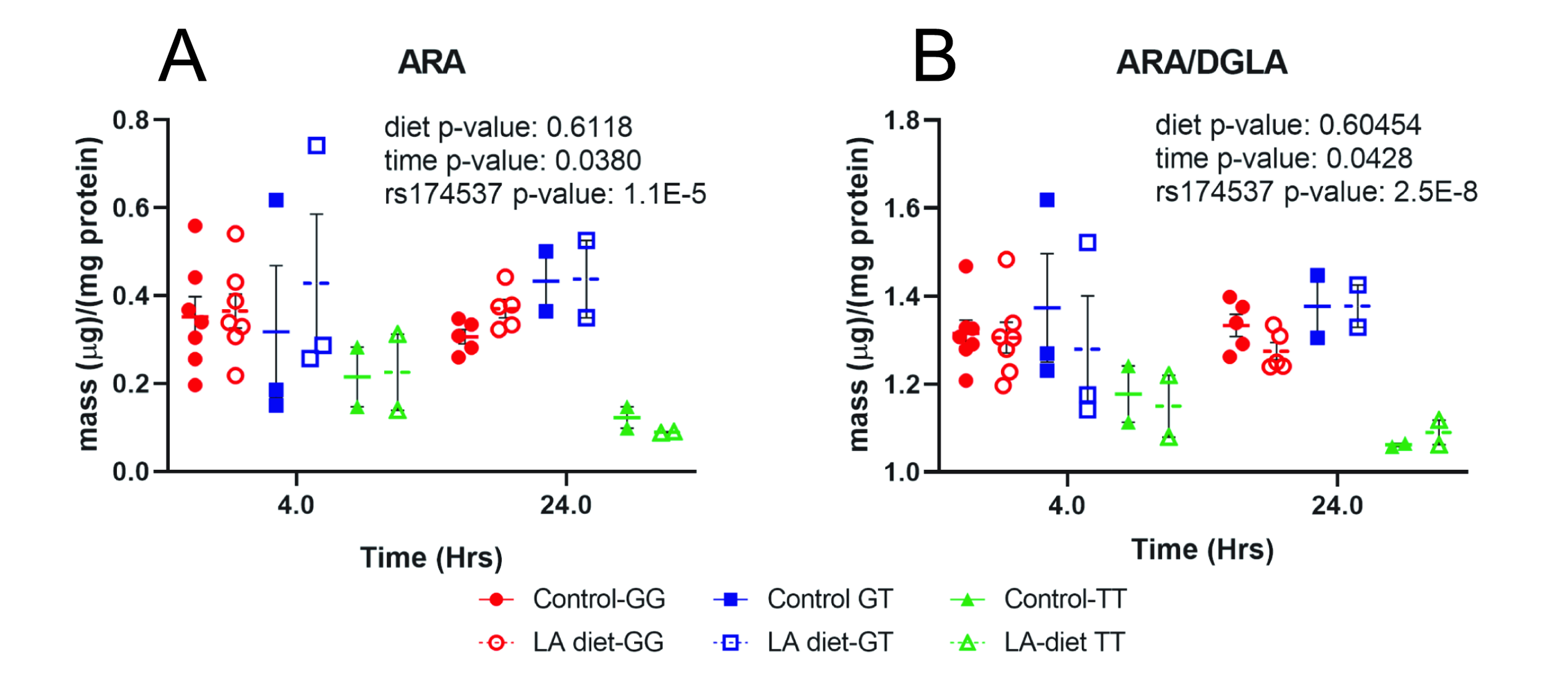

Supplement: S4 Fig — Exposure to dietary LA did not result in significantly different differences within each genotype. However, TTs consistently exhibited lower (A) ARA and (B) ARA/DGLA levels. (TIF) [file pone.0262173.s004.tif]
