# Supplementary material for: Exploiting three-dimensional human hepatic constructs to investigate the impact of rs174537 on fatty acid metabolism
Source: PLoS One. 2022 Jan 20;17(1):e0262173. doi: 10.1371/journal.pone.0262173 (PMC8775235; doi:10.1371/journal.pone.0262173)
Supplement: S1 Table — Fatty acid methyl esters (FAME) were extracted and quantified using GC-FID. Means and standard error of the mean are reported of the mass of fatty acid per 200 ul volume of media. Standard media refers to Williams-E media with FBS and contains significant amounts of ARA and other PUFAs. For the purpose of this study Control and LA media were formulated using lipoprotein deficient fetal bovine serum (FBS) to obtain a tighter control over PUFA content. (DOCX) [file pone.0262173.s005.docx]

| **S1 Table. Summary of fatty acid content of cell culture media used in this study.**  Fatty acid methyl esters were extracted and quantified using GC-FID. Means and standard error of the mean are reported of the mass of fatty acid per 200 ul volume of media. Standard media refers to Williams-E media with FBS and contains significant amounts of ARA and other PUFAs. For the purpose of this study Control and LA media were formulated using lipoprotein deficient fetal bovine serum (FBS) to obtain a tighter control over PUFA content. | | | | |
| --- | --- | --- | --- | --- |
| ***µg/200 µl*** | **Standard**  **media** | **Lipoprotein-deficient media** | **Control media** | **LA-diet media** |
| ***Myristic,***  ***C14:0*** | **0.11±0.06** | **0.06±0.04** | **--** | **--** |
| ***Palmitic,***  ***C16:0*** | **1.42±0.06** | **0.87±0.06** | **1.31±0.23** | **0.97±0.13** |
| ***Stearic,***  ***C18:0*** | **1.41±0.13** | **0.88±0.05** | **1.19±0.30** | **0.87±0.19** |
| ***Oleic,***  ***C18:1n-9c*** | **0.73±0.02** | **0.50±0.02** | **1.42±0.16** | **1.18±0.22** |
| ***LA,***  ***C18:2n-6*** | **0.25±0.00** | **0.24±0.01** | **0.60±0.01** | **2.39±0.09** |
| ***GLA,***  ***C18:3n-6*** | **--** | **--** | **--** | **--** |
| ***ALA,***  ***C18:3n-3*** | **--** | **--** | **--** | **--** |
| ***DGLA,***  ***C20:3n-6*** | **0.08±0.01** | **--** | **--** | **--** |
| ***ARA,***  ***C20:4n-6*** | **0.35±0.00** | **--** | **--** | **--** |
| ***EPA,***  ***C20:5n-3*** | **--** | **--** | **--** | **--** |
| ***Adrenic,***  ***C22:4n-6*** | **0.18±0.00** | **--** | **--** | **--** |
| ***DPA,***  ***C22:5n-3*** | **0.09±0.00** | **--** | **--** | **--** |
| ***DHA,***  ***C22:6n-3*** | **--** | **--** | **--** | **--** |
